# Study of the curative effect of Zhang’s Xibi formula and its underlying mechanism involving inhibition of inflammatory responses and delay of knee osteoarthritis

**DOI:** 10.1186/s13018-023-04453-6

**Published:** 2023-12-14

**Authors:** Qi Sun, Hongting Jin, Wuyin Li, Peijian Tong, Wenhua Yuan

**Affiliations:** 1 Institute of Orthopedics and Traumatology of Zhejiang Province, The First Affiliated Hospital of Zhejiang Chinese Medical University (Zhejiang Provincial Hospital of Chinese Medicine), Zhejiang Chinese Medical University, Hangzhou, China; 2 Fuyang TCM Hospital of Orthopedics Affiliated to Zhejiang, Chinese Medical University (Hangzhou Fuyang Hospital of Orthopedics of Traditional Chinese Medicine), Hangzhou, China; 3 Department of Orthopedic, Luoyang Orthopedic Hospital of Henan Province, Luoyang, China

**Keywords:** Knee osteoarthritis, Zhang’s Xibi formula, Immunity, Network pharmacology

## Abstract

**Objective:**

To verify the clinical efficacy of Zhang’s Xibi formula (ZSXBF) and explain the mechanism underlying its therapeutic effect.

**Methods:**

Preliminary elucidation of the clinical efficacy of ZSXBF in treating KOA in self-control studies, exploration of its mechanism of action with network pharmacology methods, and validation in animal experiments.

**Results:**

In clinical studies, ZSXBF administration effectively improved patient quality of life and reduce pain. Network pharmacology was used to explore the possible mechanisms underlying its treatment effect, and after verification in clinical experience and animal experiments, it was found that ZSXBF regulated the expression of immune-related proteins such as IL-17, ERK1, and TP53 in mouse knee joints.

**Conclusion:**

ZSXBF, which is a traditional Chinese medicine compound that is used to clear heat and detoxify, can effectively improve the clinical symptoms of KOA patients, and its underlying mechanism includes the regulation of human immune-related proteins.

**Supplementary Information:**

The online version contains supplementary material available at 10.1186/s13018-023-04453-6.

## Introduction

Osteoarthritis (OA) is a major cause of pain and disability worldwide [1, 2]. OA is an age-related joint degenerative disease, and it is the main cause of pain and dysfunction in elderly individuals. Following the development of pathophysiological concepts, OA is no longer considered a cartilage-limited disease but rather a multifactorial disease that affects the entire joint. According to the Global Burden of Disease Study 2019, 344 million people worldwide suffer from OA, and this number has increased by 114% since 1990 [3].

At present, there is no definite and effective drug for the treatment of OA, and its clinical treatment mainly involves symptomatic treatment, for example, the use of nonsteroidal anti-inflammatory drugs (NSAIDs) to relieve pain; however, when NSAID treatment is ineffective, there are almost no drugs available for follow-up treatment [4]. The lack of effective drugs to prevent and treat disease progression is still a major challenge in the treatment of osteoarthritis. Traditional Chinese medicine (TCM) has a long history of application in Asia and is known for its good clinical efficacy and safety [5, 6]. The treatment of OA with TCM is widely studied.

Zhang’s Xibi formula (ZSXBF) is the result of many years of experience in clinical practice by a famous teacher of traditional Chinese medicine in Zhejiang Province. This compound prescription, which is mainly composed of traditional Chinese medicine that is used to clear away heat and toxic materials, is considered to exert good effects in the treatment of KOA. It is generally believed that the heat-clearing and detoxification functions of this medicine may be related to the inhibition of immune responses that are caused by inflammation in the human body [7–9].

However, the components of ZSXBF and the mechanism underlying its treatment effects against KOA have not yet been elucidated. Therefore, this study aimed to predict the key active ingredients and potential pharmacological mechanisms of ZSXBF in the treatment of OA through clinical experience, network pharmacology, and animal experimental methods.

## Methods and materials

### Clinical retrospective study

This study was approved by the Ethics Committee of Hangzhou Fuyang Traditional Chinese Medicine Bone Injury Hospital (No. 2023-LW-LC-003).

Data from 146 KOA patients who were treated with ZSXBF at Fuyang Bone Injury Hospital in Hangzhou City between January 1, 2021, and December 31, 2021, were collected. To evaluate clinical outcomes, visual analog scale (VAS) and Leqiesne’s score examinations were performed one week before (baseline) and one month after treatment.

ZSXBF consists of 6 Chinese herbs, including Dang-gui (DG), Chuan-niuxi (CNX), Ze-lan (ZL), Huang-bo (HB), Tu-fuling (TFL) and Mao-rensheng (MRS). The detailed data of ZSXBF are shown in Table 1. All of the patients who were enrolled underwent more than 1 course of ZSXBF treatment.

**Table 1:** The composition of ZSXBF

Herb	Latin name	Weight(g)	Parts used
Danggui	*Angelica sinensis*	6	Root
Chuanniuxi	*Cyathula officinalis*	15	Root
Zelan	*Lycopi herba*	15	Stem
Huangbo	*Phellodendron amurense*	5	Bark
Tufuling	*Smilax glabra*	25	Root
Maorensheng	*Actinidia macrosperma*	30	Root

### Network pharmacological analysis of ZSXBF

The chemical components of the traditional Chinese medicine herbs in ZSXBF were searched in the Traditional Chinese Medicine Systems Pharmacology Database and Analysis Platform (TCMSP) databases. Oral bioavailability (OB) and drug likeness (DL) were set as the screening criteria. In addition, eligible putative targets of these potential active compounds were identified in the TCMSP databases.

Using knee osteoarthritis as the key word, associated target genes were identified by a joint search of the Genecards, Online Mendelian Inheritance in Man (OMIM), PharmGKB, Therapeutic Target Database (TTD), and DrugBank databases. All the collected target genes, which were in the form of abbreviations, were edited in the same Microsoft Excel table, and duplicates were removed.

R was used to screen target genes that were shared by KOA and ZSXBF. Then, the STRING online tool was used to analyze the target genes to generate a protein‒protein interaction network (PPI). The enrich plot tool was used for Gene Ontology (GO) analysis and Kyoto Encyclopedia of Genes and Genomes (KEGG) pathway analysis.

To analyze the binding affinities and modes of interaction between the drug candidate and their targets, AutodockVina 1.2.2, a silico protein–ligand docking software was employed [10]. The molecular structures of quercetin was retrieved from PubChem Compound (https://pubchem.ncbi.nlm.nih.gov/) [11]. The 3D coordinates of TP53 (PDB ID, 6VTC; resolution, 1.83 Å) was downloaded from the PDB (http://www.rcsb.org/pdb/home/home.do). For docking analysis, all protein and molecular files were converted into PDBQT format with all water molecules excluded and polar hydrogen atoms were added. The grid box was centered to cover the domain of each protein and to accommodate free molecular movement. The grid box was set to 30 Å × 30 Å × 30 Å, and grid point distance was 0.05nm. Molecular docking studies were performed by Autodock Vina 1.2.2 (http://autodock.scripps.edu/).

### Preparation of ZSXBF

All the botanical drugs in ZSXBF were provided by the First Affiliated Hospital of Zhejiang Chinese Medical University (Hangzhou China). After soaking in 10 volumes of distilled water for 1 h, DG, CNX, ZL, HB, TFL, and MRS were mixed (the total dry weight was 1 kg) at a ratio of 6:15:15:9:25:30 for reflux extraction (three times, 1.5 h/time). Then, the solution was concentrated (3 g crude drug/mL).

### Chemical components of ZSXBF and quality control

To determine the main chemical components of ZSXBF, high-performance liquid chromatography (HPLC) was used. We added 0, 1 ml, 2 ml, and 4 ml of ZSXBF soup to four round-bottomed flasks and added 20 ml of 70% methanol (TEDIA, NO. 21015240) solution. After weighing, the solution was ultrasonicated for 30 min, allowed to cool, and weighed again, and the extraction solvent was used to make up the lost weight. The mixture was shaken well and filtered, and the filtrate was taken to obtain the final product.

An Ultimate ®UHPLC AQ-C18 (1.8 μm, 2.1 × 100 mm column) chromatographic column was used. The mobile phase consisted of acetonitrile (TEDIA, No. AS1122–801) and 0.2% acetic acid (ACROS ORGANICS, No. A0365737). The gradient elution procedure is shown in Table 2. Chromatographic analysis was performed at 35 ℃ with a flow rate of 0.30 ml/min and an injection volume of 5 μl.

**Table 2:** Gradient elution procedure

Time (min)	Mobile phase (A%)	Mobile phase (B%)
0	5	95
2	8	92
25	25	75
33	40	60
35	70	30
38	5	95

**Fig. 1:**
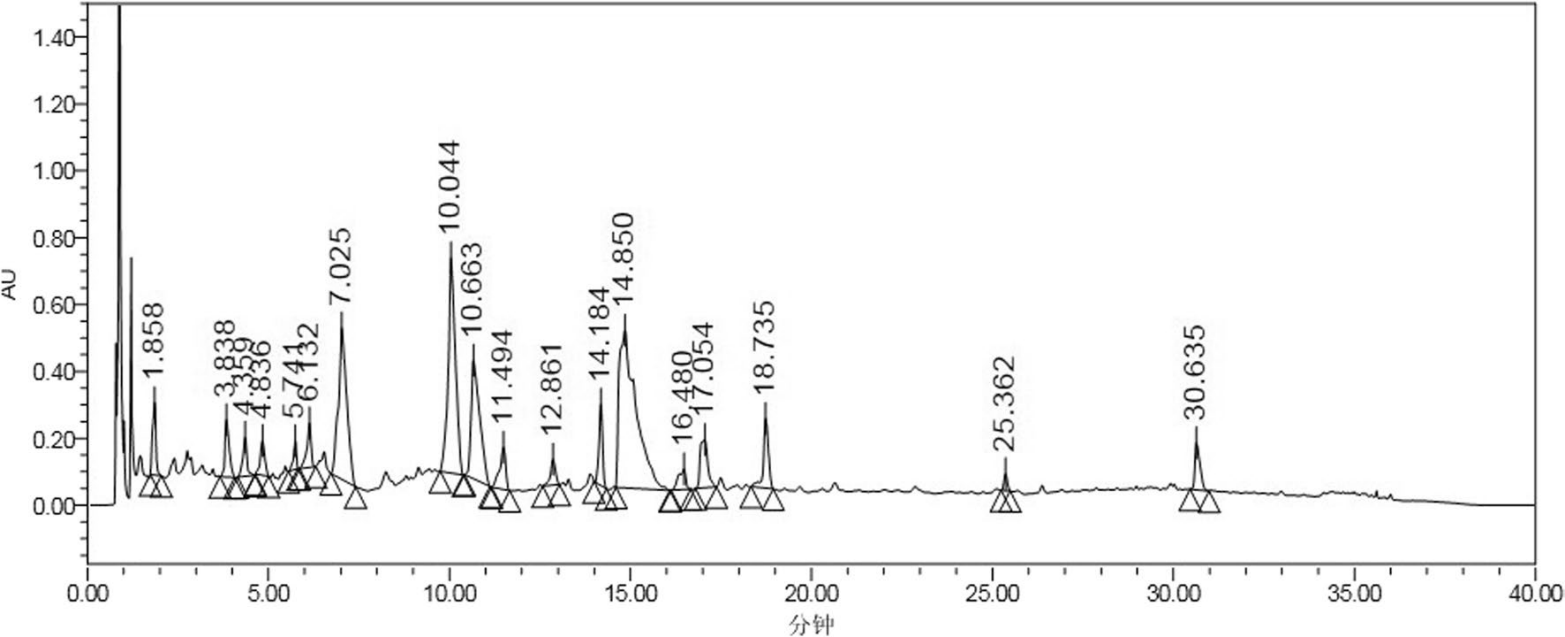
Results of UPLC analysis of ZSXBF. All chromatographic peak signals were recorded at 280 nm, and the peak area was integrated according to the instrument’s protocol. The components in the main peak were beta-sitosterol, stigmasterol, betavulgarin, rubrosterone, quercetin, berberine, coptisine, kihadalactone A, obacunone, delta 7-stigmastenol, dehydrotanshinone II A, phellopterin, delta7-dehydrosophoramine, dihydroniloticin, niloticin, worenine, thalifendine, and rutaecarpine

The botanical medicinal materials used in this study were sent to the Drug Testing Laboratory of Zhejiang University of Traditional Chinese Medicine Academy of Traditional Chinese Medicine for identification according to The Chinese Pharmacopoeia (2020 edition). The quality of ZSXBF was controlled, and the exact chemical composition was determined by ultra-performance liquid chromatography (UPLC). UPLC identified 18 chromatographic peaks representing 18 drug monomers (Fig. 1).

### Experimental animals

Destabilization of the medial meniscus (DMM) was performed on 10-week-old male C57BL/6 mice to establish the knee osteoarthritic model. Briefly, DMM surgery was performed on the right hind limbs of C57BL/6 mice. First, a 3 mm longitudinal incision was made on the medial part of the knee under anesthesia, and blunt dissection of the knee extensor muscles and patellar ligament (MMTL) was performed. Then, the MMTL was transected to destabilize the medial meniscus. Finally, the medial joint capsule was sutured, and the skin was closed. Sham surgery was also performed on C57BL/6 mice with a similar surgical approach but without manipulating the joint tissues.

All the C57BL/6 mice were randomly divided into three groups (*n* = 6 in each group): the sham group, DMM group, and XBF group. In the XBF group, the drug was orally administered to the mice for 8 consecutive weeks (0.2 ml/10 g body weight, once a day) beginning on the day after DMM surgery.

C57BL/6 mice were purchased from the Experimental Animal Center of Zhejiang Chinese Medical University (Hangzhou, China). All the studies were approved by the Animal Ethics Committee of Zhejiang Chinese Medical University (20200622-07).

### Microcomputed analyzes

All the mice were sacrificed at 8 weeks after surgery, and the right knee joints were collected. Then, microcomputed tomography (μ-CT) was used to analyze the knee joints. The area between the proximal tibia growth plate and the tibial plateau was chosen as the region of interest, and the parameters collected by μ-CT were percent bone volume (BV/TV, %), trabecular thickness (Tb. Th, mm), and bone mineral density (BMD, g/cm^3^).

### Histological analysis and immunohistochemistry

After μ-CT analysis, the samples were successively fixed in 4% paraformaldehyde for 72 h, decalcified with 14% ethylene diamine tetra acetic acid (EDTA) solution for 14 days, and embedded in paraffin. Then, 3 μm thick sections of the medial compartment of the joints were generated for Alcian Blue Haematoxylin/Orange G (ABH) staining to analyze gross cartilage structural changes. The thickness of the tibial cartilage was scored by three blinded observers.

Immunohistochemistry (IHC) was performed to observe the expression of type II collagen (Col2, 1:300 in PBS), matrix metallopeptidase 13 (MMP13, 1:300 in PBS), interleukin 17 (IL-17, 1:300 in PBS), tumor protein p53 (TP53, 1:300 in PBS), mitogen-activated protein kinase 14 (MAPK14, 1:200 in PBS), and extracellular regulated protein kinase 1 (ERK1, 1:200 in PBS).

## Results

### Clinical evaluation of ZSXBF efficacy in the treatment of KOA

The baseline characteristics of the participants in the incidence and progression cohorts are shown in Table 3.

**Table 3:** Baseline characteristics of participants and knees in the incidence cohort

	Participants (*N* = 146)
Age (years)	58.06 ± 9.275
Sex (*n*, %)	
Male	86, 58.9
Female	60, 41.1
KOA (*n*, %)	
I–II	99, 67.8
III–IV	47, 32.2
Osteophyte size (n, %)	
1	25, 17.1
2	76, 52.1
3	33, 22.6
4	11, 7.5

To evaluate the clinical efficacy of ZSXBF in the treatment of KOA, we scored the treatment outcomes of 146 patients. After treatment with ZSXBF, the VAS scores and Lequesne scores were significantly improved, indicating that after treatment with ZSXBF, pain levels improved, and walking and ability to perform activities of daily life improved (Table 4).

**Table 4:** Evaluation of clinical efficacy of ZSXBF in the treatment of KOA

	Before (${\overline{\text{x}}} + {\text{s}}$)	After (${\overline{\text{x}}} + {\text{s}}$)	*P*
VAS	6.2 ± 1.295	2.87 ± 0.849	0.000
*Lequesne*			
Pain or discomfort	4.46 ± 1.536	2.5 ± 0.841	0.000
Walking ability	5.27 ± 1.086	3.62 ± 0.706	0.000
Ability to perform activities of daily life	4.02 ± 1.374	2.71 ± 0.892	0.000

### ZSXBF reversed the KOA phenotype in DMM model mice.

**Fig. 2:**
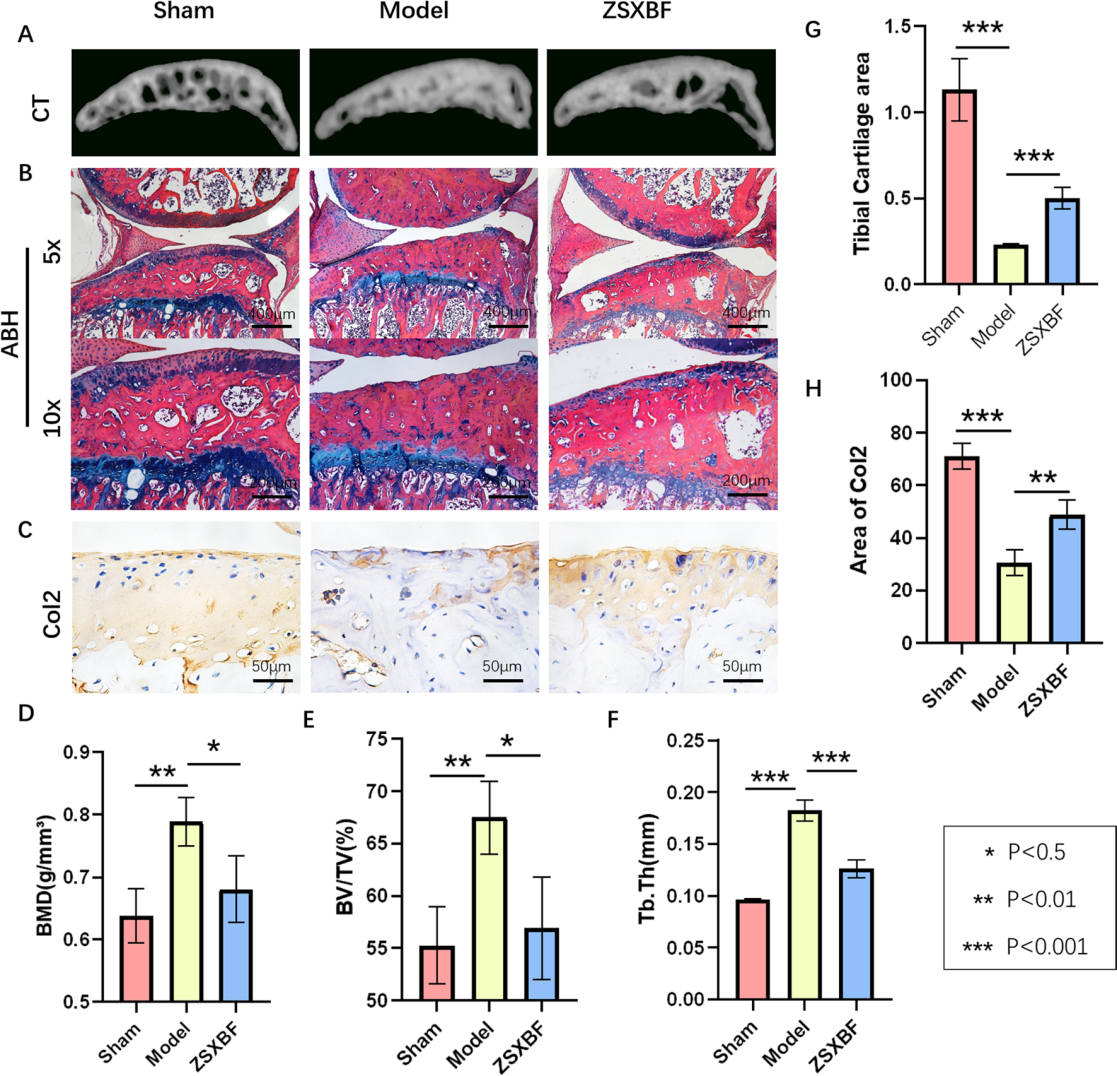
ZSXBF inhibits osteoarthritis. **A** Representative μCT images. Quantification of microstructural parameters, including **D** BMD, **E** BV/TV, and **F** Tb.Th. **B** Alcian blue and haematoxylin/orange G staining of the knee joint. **G** Tibial cartilage area. **C**, **H** Representative IHC images and quantification of Col2. Scale bar = 50 μm

To prove the effect of ZSXBF treatment on cartilage and subchondral bone, which are the sites of injury in KOA, DMM surgery was performed on C57BL/6J mice, and ZSXBF was intragastrically administered for 8 weeks. After 8 weeks, knee joint tissue samples were taken for μ-CT scanning, and 3D reconstruction was performed to obtain representative images of the knee joints. The μ-CT scanning results showed that the DMM model group had significant subchondral bone sclerosis and cartilage loss and that the BMD, subchondral bone BV/TV, and Tb.Sp values were significantly different (Fig. 2A, D, E). Additionally, the tibial cartilage area and the expression of Col2 were decreased (Fig. 2C, H). Moreover, ZSXBF treatment reversed the trends observed in the cartilage and subchondral bone (Fig. 2B, G).

### Active compounds in ZSXBF and target prediction

**Fig. 3:**
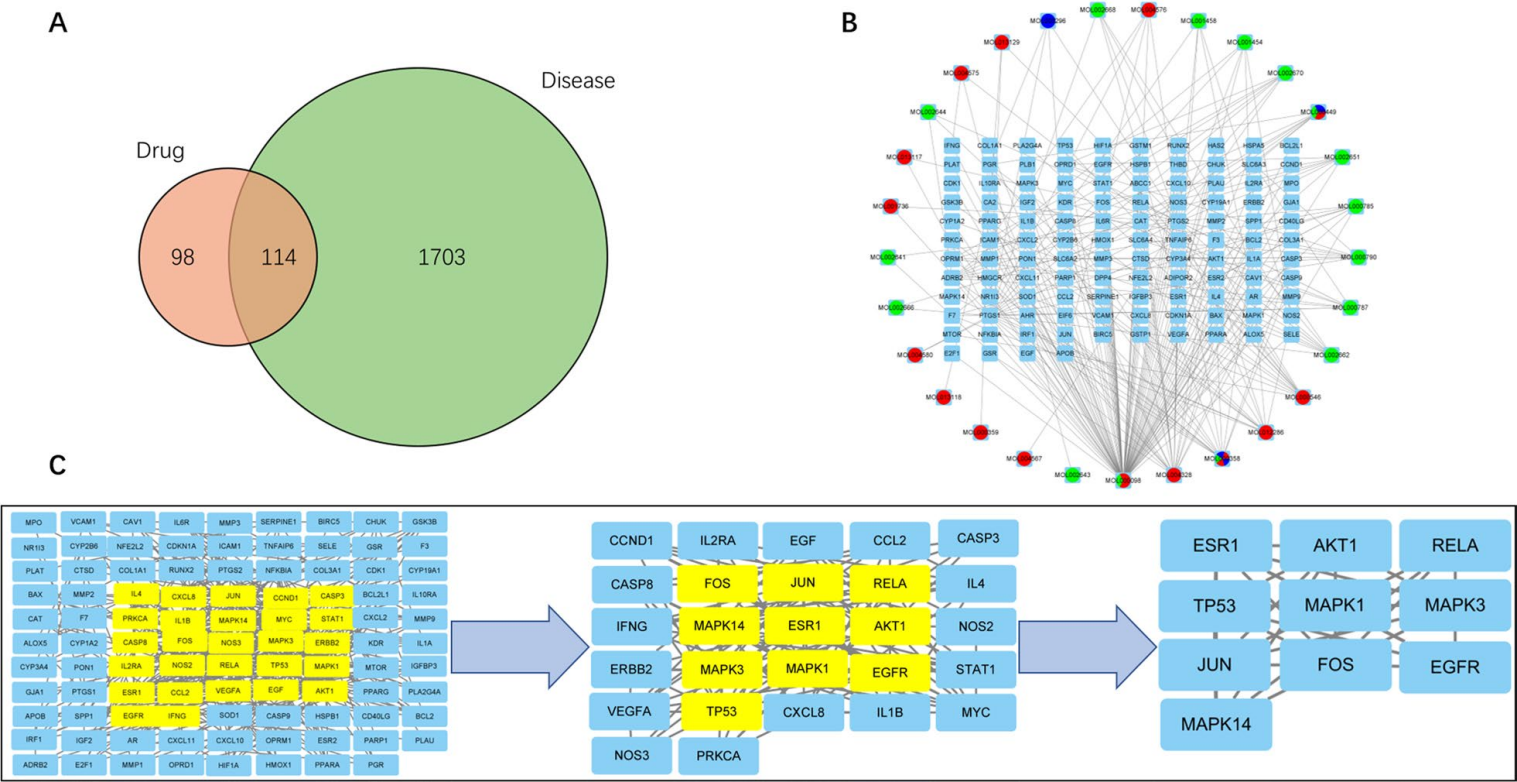
Drug-target-disease network and PPI network construction. **A** The intersection of ZSXBF-related targets and KOA-related targets. **B** The target-disease network includes 29 active components and 114 target genes. Circles represent active components, and rectangles represent related targets. **C** PPI network of predicted targets of ZSXBF in the treatment of KOA

The Pharmacology Database and Analysis Platform database were used in this analysis. Six drugs in ZSXBF were identified in the TCMSP, and the TCMSP showed that CNX, DG, HB, TFL, ZL and MRS had 4, 2, 37, 15, 2 and 0 targets, respectively. After eliminating duplicate targets, 212 predicted target genes were identified. To determine the potential targets of OA, we searched 1817 OA-related target genes in the GeneCards, DrugBank, TTD, OMIM, and PharmGkb databases. To investigate the potential target genes of ZSXBF in the treatment of OA, Venn analysis was performed, and 114 genes were identified as overlapping genes between ZSXBF and OA targets (Fig. 3A).

We used STRING to construct a PPI network to determine the key proteins in the treatment of OA with ZSXBF. The thresholds of betweenness, closeness, degree, eigenvector, information, LAC and subgraph were set in two steps based on the topology parameters calculated by Cytoscape. Finally, ten key proteins, including ESR1, AKT1, RELA, TP53, MAPK1, MAPK3, JUN, FOS, EGFR, and MAPK14, were identified (Fig. 3B, C).

**Fig. 4:**
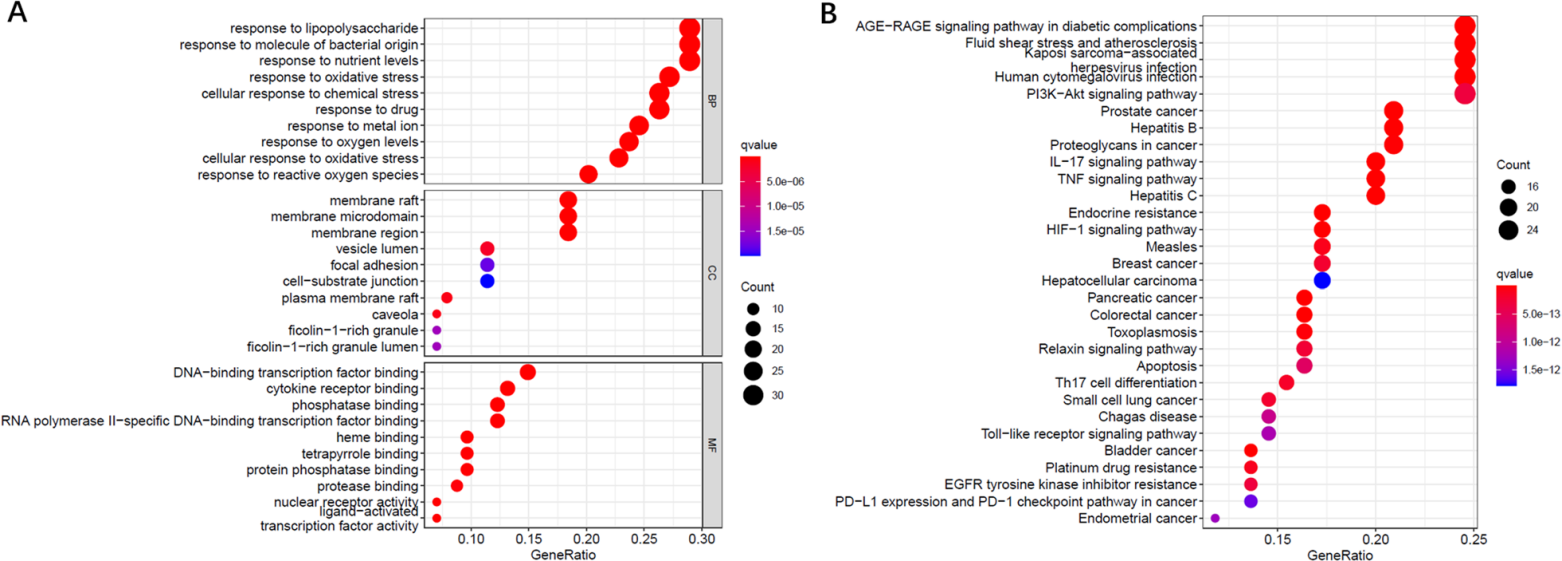
GO and KEGG functional analyzes. **A** The top 10 GO enrichment terms in biological processes, cellular components, and molecular function. **B** The top 30 enriched KEGG signaling pathways

GO and KEGG pathway enrichment analyzes of 168 tandem targets were conducted with the database. Based on network pharmacological analysis, we found that the AGE-RAGE signaling pathway and PI3K-Akt signaling pathway may be the most relevant signaling pathways for the treatment of OA with ZSXBF (Fig. 4A, B).

To evaluate the affinity of the drugs in ZSXBF for their targets, we performed molecular docking analysis. The binding poses and interactions of the main drug candidate with the main protein were obtained with Autodock Vina v.1.2.2 (Additional file 1: Fig. S1). Results showed that drug candidate bound to its protein targets through visible hydrogen bonds and strong electrostatic interactions. Moreover, the hydrophobic pockets of target was occupied successfully by the candidate drug. For TP53, quercetin had low binding energy of -9.784 kcal/mol, indicating highly stable binding.

### Validation of the mechanism underlying the therapeutic effect of ZSXBF in DMM mice

**Fig. 5:**
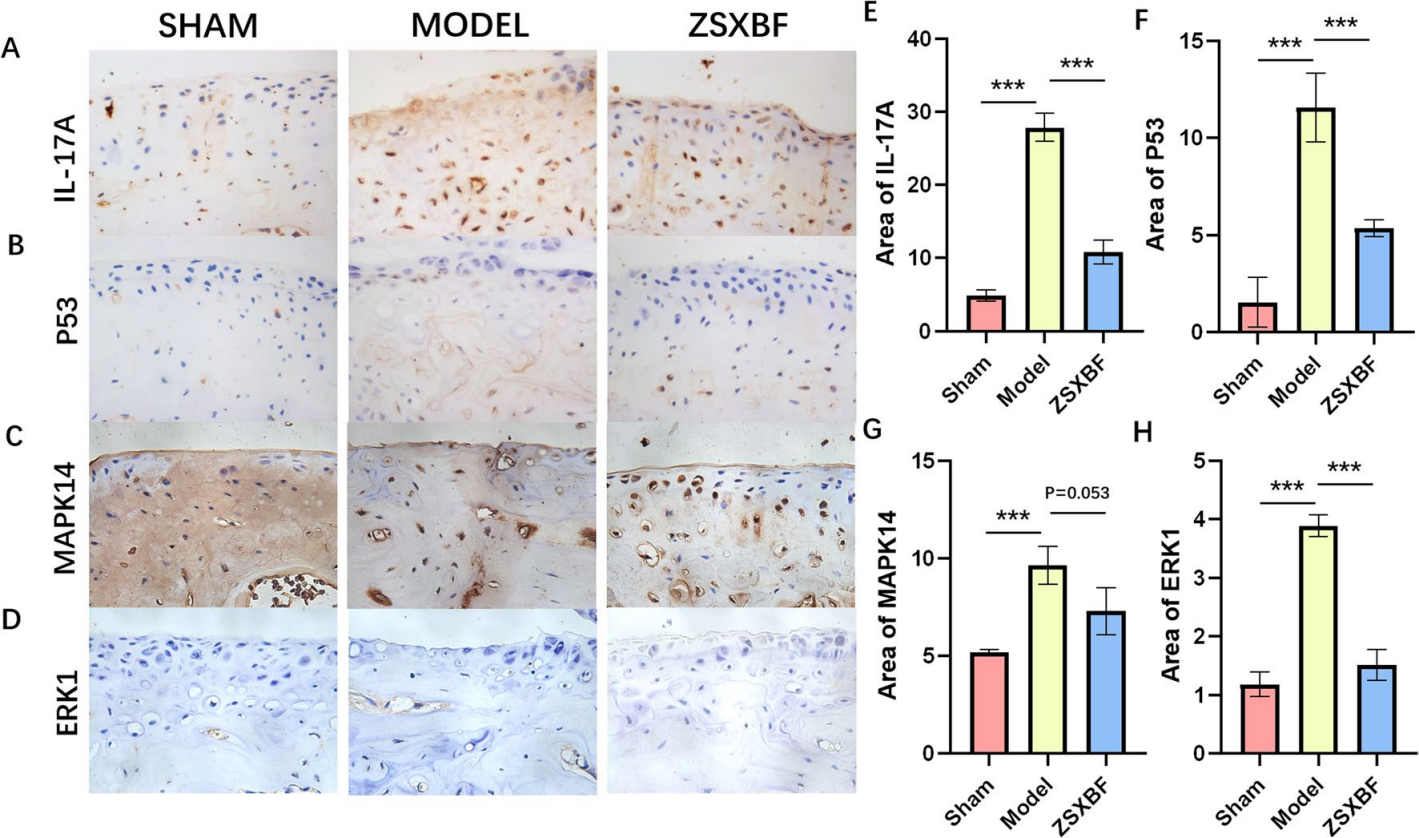
ZSXBF regulates targets of cartilage degeneration **A**–**H** Representative IHC images and quantification of IL-17A, TP53, MAPK14, and ERK1 in the chondro-osseous region. *P < 0.05, **P < 0.01, ***P < 0.001

The network pharmacology results suggest the possible mechanism by which ZSXBF treats KOA. Combined with our understanding of ZSXBF TCM treatment, we aimed to verify whether ZSXBF regulates cellular immunity, and we performed IHC staining for IL-17A, TP53, MAPK14, and ERK1. The results showed that the expression levels of IL-17A, TP53, MAPK14 and ERK1 in the articular cartilage of DMM model mice were significantly higher than those in control mice, suggesting that the pathogenesis of knee osteoarthritis is closely related to the immune factor IL-17. The expression levels of IL-17A, TP53 and ERK1 in the treatment group were significantly lower than those in the DMM model group, and there was no significant difference in the expression level of MAPK14 (Fig. 5A–H).

## Discussion

TCM is an ancient treatment method, and it has been widely accepted and applied in the treatment of knee osteoarthritis [12, 13]. However, since it is a treatment concept based on experience, clinical and laboratory efficacy evaluation should play a more important role in TCM treatment development. In previous experience, ZSXBF was thought to exert an effective therapeutic effect on knee osteoarthritis, but evaluation of the specific effects and mechanism of action were not fully completed.

Before verifying the mechanism, our study further elucidates the clinical efficacy of ZSXBF. Through a retrospective analysis of the clinical efficacy of ZSXBF, the agreement party, we found that ZSXBF can effectively relieve the pain of KOA patients and improve their quality of life according to Lequesne’s score. In this retrospective study, patients' own baseline levels were used as the control group.

To compensate for the limitations of the self-control study, we further verified the therapeutic effect of ZSXBF in experimental animals in a random control study. The DMM model is a widely used and recognized mouse model of KOA [14, 15]. We verified the therapeutic effect of ZSXBF on knee osteoarthritis in DMM model mice, and the mechanism included inhibiting subchondral bone sclerosis and delaying chondrocyte degeneration.

Then, the mechanism underlying the therapeutic effect of ZSXBF was analyzed through network pharmacology. In contrast to chemical drugs, TCM has complex components, and it has multiple effective compounds and targets. In recent years, a new method has been used to study the mechanism underlying drug treatment of complex diseases [16]. Furthermore, this approach, known as network pharmacology, has also been used to study the mechanism of action of complex drugs, such as TCM. These studies have explored the potential mechanisms by which TCM compounds treat diseases through the application of network pharmacology [17–19].

Through network pharmacology, we identified possible therapeutic targets of ZSXBF. These therapeutic targets were compiled and calculated from other researchers' studies on TCM components and KOA through a computer system. Some researchers studied CNX and found that it can protect against chondrocyte damage induced by IL-1β [20]. Research on DG has shown that it can play a therapeutic role in some immune diseases, such as immune-mediated aplastic anemia, which is a cause of osteonecrosis [21, 22].

Changes in the T-cell population have been observed in KOA, including an increase in the proportion of Th17 cells [23]. A study has shown that inhibiting the expression of the Th17 cell-related factor IL-17 in damaged chondrocytes can reduce joint deformation and delay cell Aging[ [24]. TP53, which was first discovered as a tumor suppressor, plays an important role in cell cycle regulation, including the regulation of cellular senescence and apoptosis [25]. The regulation of the cell cycle by TP53 occurs in many cell types in humans, including in chondrocytes [26]. Dysregulation of TP53 has been shown to inhibit the production of IL-17 [27]. During inflammation, IL-17 is also involved in the regulation of ERK1 [28]. In our experiments, the use of ZSXBF improved the expression of IL-17, TP53 and ERK1 in chondrocytes. KEGG enrichment results showed that ZSXBF can affect the progression of KOA by regulating IL-17-related signaling pathways, which is consistent with our experimental results. Quercetin, a significant pharmaceutical candidate, has been identified in CNX, HB, and TFL. Our investigation reveals that quercetin exhibits binding affinity toward TP53, facilitated by visible hydrogen bonds and robust electrostatic interactions.MAKP14 is another target that is important in the progression of KOA [29]. In the PPI, MAPK14 was the core target of ZSXBF in the treatment of KOA. However, in the animal experiment results, the regulatory effect of ZSXBF on MAPK14 lacked statistical significance. This result may have occurred due to the insufficient sample size in the animal experiments. may be limited by the existing research progress and the complexity of traditional Chinese medicine compatibility, resulting in the need for further experimental verification.

In summary, in this article, we have preliminarily discussed the therapeutic effect and underlying mechanism of Zhang's prescription in the treatment of knee arthralgia. We believe that ZXBF has a clear therapeutic effect in clinical practice. After computer data analysis and experimental verification, we believe that the mechanism by which ZSXBF treats OA is related to IL-17, ERK1 and TP53. Combined with the published results of other researchers, we speculate that ZXBF can delay the progression of KOA by regulating the IL-17-mediated immune response.

### Supplementary Information

**Additional file 1: Fig. S1**. Binding mode of screened drugs to their targets by molecular docking. Binding mode of quercetin to TP53.

## Data Availability

All data generated during this study are included in this manuscript.
