# Supplementary figures and images for: Study of the curative effect of Zhang’s Xibi formula and its underlying mechanism involving inhibition of inflammatory responses and delay of knee osteoarthritis
Source: J Orthop Surg Res. 2023 Dec 14;18:963. doi: 10.1186/s13018-023-04453-6 (PMC10722826; doi:10.1186/s13018-023-04453-6)

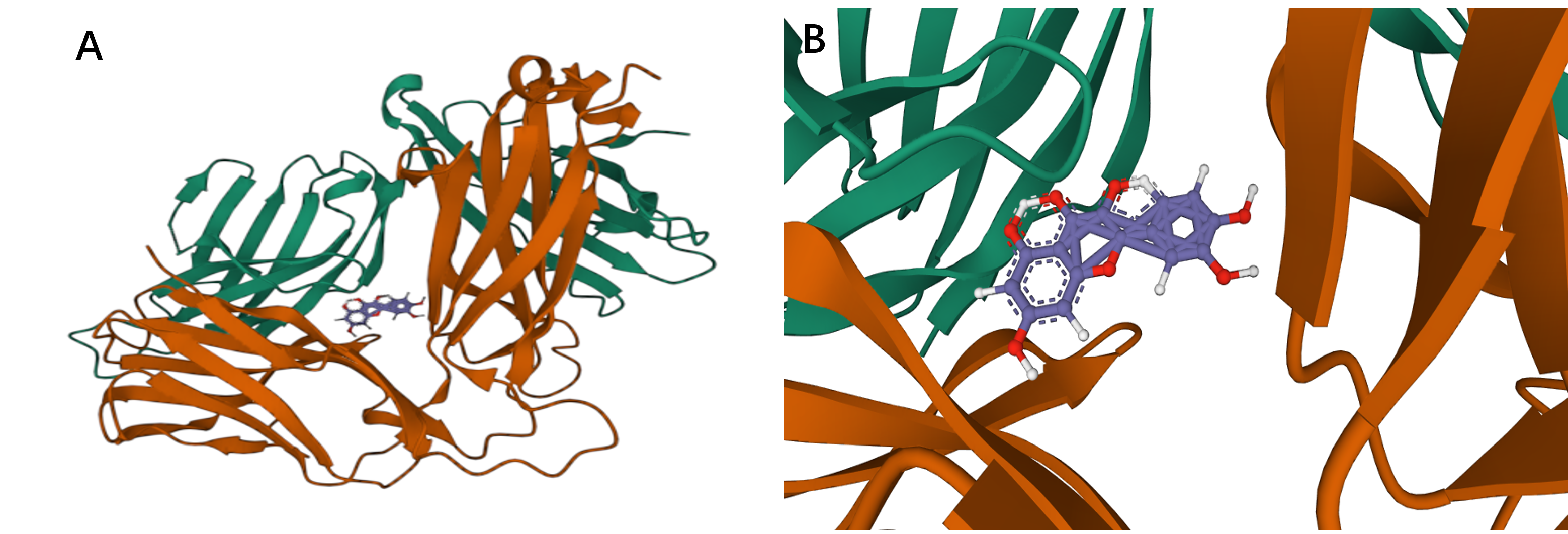

Supplement: Supplementary file 1 — Additional file 1: Fig. S1. Binding mode of screened drugs to their targets by molecular docking. Binding mode of quercetin to TP53. [file 13018_2023_4453_MOESM1_ESM.tif]
